# Mainstream and Special School Attendance among a Dutch Cohort of Children with Down Syndrome

**DOI:** 10.1371/journal.pone.0091737

**Published:** 2014-03-17

**Authors:** Jacobus P. van Wouwe, Helma B. M. van Gameren-Oosterom, Paul H. Verkerk, Paula van Dommelen, Minne Fekkes

**Affiliations:** 1 Department of Child Health, Netherlands Organization for Applied Scientific Research TNO, Leiden, the Netherlands; 2 Department of Life Style, Netherlands Organization for Applied Scientific Research TNO, Leiden, the Netherlands; University Children's Hospital Tuebingen, Germany

## Abstract

**Object:**

To determine the level of mainstream education in a nationwide cohort of adolescents with Down Syndrome (DS), and to find characteristics related to mainstream or special school attendance.

**Method:**

Dutch children with DS born in 1992, 1993 or 1994, were assessed when 16–19 years old. Parents scored school enrolment between the age of 4–18 years, general characteristics and the levels of intellectual disability using the Dutch Social Competence Rating Scale. Associations between disability and years in mainstream school were assessed by ordinal logistic regression, adjusting for sex and parental education.

**Results:**

We collected data from 170 boys and 152 girls (response 63%); mean age 18.3 years (ranges 16.8–19.9). Intellectual disability was mostly moderate (43%). Most children (74%) entered mainstream education between 4 and 6 years of age. At 13 years 17% was in mainstream school and 7% stayed in up to 16 years. From the age of 8 years onwards the majority was in special education, while 6% never attended school. Girls were more often in mainstream school and stayed in longer. Level of disability was significantly associated with number of years in mainstream education.

**Conclusion:**

Three out of four Dutch children with DS entered mainstream primary education, however late entry and high dropout are common.

## Introduction

Down Syndrome (DS] is the most common genetic cause of intellectual disability. In the Netherlands, 245 infants with DS are live born annually [1] Their growth and development follow a specific pattern and an array of treatable concomitant medical conditions is recognized. Considerable efforts focus on early recognition and treatment of these conditions in order to guarantee their optimal health, growth and development, especially their motor and cognitive development [2]. In an era where survival has positively changed and these medical conditions are managed, social competence and functioning are factors to focus on now.

One of these issues is enrolment in mainstream education, as part of the increased focus on social participation. In the Netherlands, the Dutch Down Syndrome Foundation (SDS, parent organization) advocates a pro-active attitude and assists to communicate the special needs towards schools. Only a few studies focus on the advantages of mainstream vs. special education in children with DS. Achievements of teenagers with DS in mainstream education may improve some short term progress for communication, expressive language or literacy and reading skills [3]. Another study shows that mainstream education has a modest beneficial effect on academic scores, independently of the level of intellectual disability, although no comparison with special education was made [4]. A Dutch study showed that children with DS in mainstream education learn more academics, most pronounced for reading skills. However these advantages were also determined by other factors, such as parental educational level [5].

It is estimated that 56% of all Dutch children born since the 1990s start in mainstream school, and that 22% are still in at the age of 12 years [6]. In other countries the proportion of children entering mainstream education and staying in increased over the years [7]. In Norway nowadays it is common for children with DS to enter mainstream education [8]. The actual overall schooling (getting in and staying) of children with DS has not been studied in a nationwide cohort before. Therefore, in the present study we aim to investigate the actual school career in adolescents with DS nationwide and to find characteristics related to those in mainstream or special school. Hereby we focus on two issues: which type of schools have they visited and for how many years, and are cognitive disability and sex associated with type of education?

## Methods

### Data Collection

Data were collected from a nationwide cohort assessed at the age of 16–19 years [9]. This included children born in 1992, 1993, and 1994, living in the Netherlands. All known parents (n = 513) received a written request by the parent organization (SDS); no selection was made. Approximately 80% of all parents of a child with DS of this age in the Netherlands had joined this organization and was therefore invited to participate. Written informed consent was obtained from parents/next of kin of all participants. Permission of an ethical committee was not obtained because it is in the Netherlands not required for this type of study with anonymous data collection

### School Enrolment

Parents were asked to score separately, the type of school their child had visited, from the age of 4 to 18 years for each year. The options were: no formal education, mainstream primary or secondary school, special primary or secondary school, other school type specified. Parents were invited to add comments. If only the name of a school was given the school type was classified by one of the authors (HvG-O). If more items were entered, the answer was counted in the category ‘combinations and unknown’.

### Cognitive Disability

Levels of cognitive disability were based on the Dutch Social Competence Rating Scale (SRZ). Its validity and reliability regarding practical daily skills in children and adults with DS has been reported to be good [10], [11]. The SRZ contains 31 items that determine the level of intellectual disability. These represent the following skills: *profound intellectual disability* (hardly able to dress oneself, wash hands and face properly and use adequate toilet hygiene, just able to eat independently without the use of a knife, and nearly unable to speak), *severe intellectual disability* (can undress, able to wash hands and face, uses knife and fork, cleans up after dinner, speaks with incomplete sentences with unclear pronunciation, understood only by close caregivers or familiar people), *moderate intellectual disability* (dresses completely, washes hands and face properly, uses adequate toilet hygiene, uses knife and fork including cutting meat without bone, able to walk outside home without supervision, and speech is mostly understood by others), *mild intellectual disability* (dresses oneself completely also footwear, keeps complete personal hygiene, sets table properly, plates, cutlery, napkins, food, etc., permitted to walk several streets away from home without supervision, uses compound sentences, speech and language understood by unfamiliar people).

### Statistical Analyses

General characteristics of the study population were determined. As our subjects varied in age between 16 and 19 year outcome measures were; the type of school at 16 years of age (special, mainstream or none), the overall presence in mainstream primary school (ever in mainstream) and the total years in mainstream and special school before the age of 17.

To evaluate differences between boys and girls, we applied *t*-tests for continuous and chi-square tests for categorical variables. Ordinal logistic regression analyses were performed to assess the association between intellectual disability and the years that children were in mainstream. Ordinal regression analysis with a complementary log-log link function was applied with the outcome measure of three categories years of enrolment in mainstream. The models were presented unadjusted and adjusted for parental education and sex. For all analyses, statistical tests were 2-tailed and significance was defined at *p*<0.05. The analyses were performed using Statistical Package for the Social Sciences, version 20.0 for Windows (SPSS Inc, Chicago, Illinois).

## Results

Cross-sectional data were obtained from the nationwide cohort. Data from 322 subjects out of the 513 invited, were suitable for analysis (response 63%). Table 1 shows the general characteristics, stratified by sex; 53% of the subjects were boys, nearly all subjects were of Dutch descent. They were on average 18 years old (18.3 years, ranges 16.8 to 19.9). The vast majority lived at home and had practiced an early intervention program, such as the adapted version of the Macquarie/Portage Programs. The level of cognitive disability was mostly moderate, 43%, however the distribution was wide with 30% severe, 10% profound and 17% mild. No differences between boys and girls were noticed in age, residence or parental education. However, the level of cognitive disability varied. More girls scored a level of mild cognitive disabilities, while relatively more boys scored severe and profound cognitive disabilities.

**Table 1:** General characteristics of the adolescents with Down syndrome (n = 322).

	Total		Boys		Girls		
*General Characteristics*	n	%	n	%	n	%	*p* *
Number of Subjects	322	100	170	53	152	47	<0.001
Dutch Descent∧	300	93	162	95	138	91	0.110
Age in Years (range)	16.8–19.9		16.9–19.9		16.8–19.8		
Age in Years (mean ± SD)	18.3±0.8		18.3±0.8		18.3±0.8		0.553
Living at Home	283	88	149	88	134	88	0.888
Early Stimulation Practiced	265	82	143	84	122	80	0.366
*Parental Education*							0.524
Low	39	12	23	14	16	11	
Medium	105	33	58	34	47	31	
High	177	55	89	52	88	58	
*Level of Cognitive Disability*							<0.001
Mild	54	17	16	9	38	25	
Moderate	139	43	73	43	66	44	
Severe	97	30	58	34	39	26	
Profound	31	10	23	14	8	5	

Abbreviation: SD – standard deviation.

* Boys with DS compared to girls with DS.

∧ Both parents born in the Netherlands.

**Figure 1:**
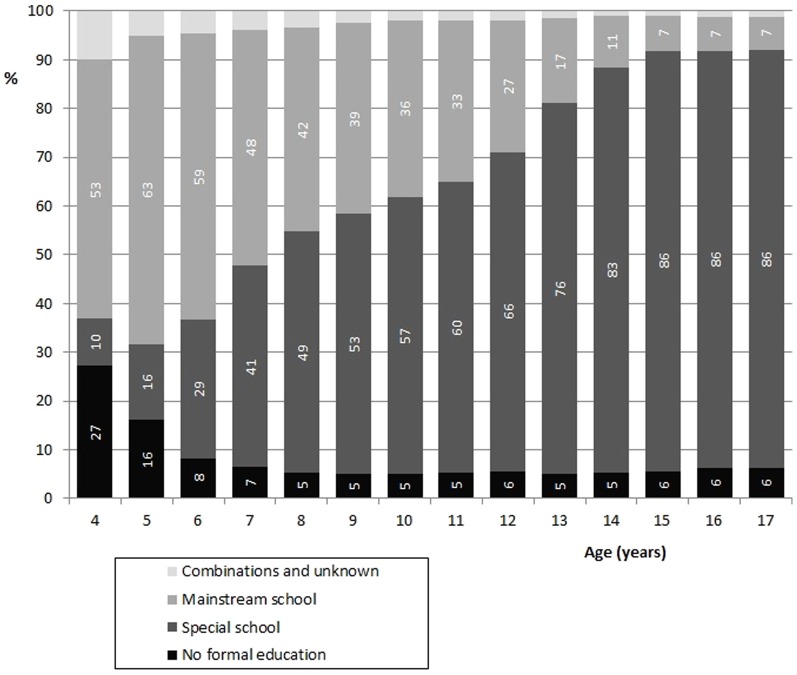
School enrolment of Dutch children with Down Syndrome by age, as retrospectively reported by their parents at the end of school career (n = 322).

The scores on actual school enrolment are summarized in Figure 1. Entry of school started between the ages of 4 and 6 years. The majority was enrolled in mainstream at the ages 4, 5, 6 and 7 years (48–63%). Dropout rate was high: from the age of 7 onwards, annually 3–6% of the children transferred out towards special education. At transition from primary to secondary school (at approximately 12–13 years), efflux was larger (6–10%). Only 17% of the children was in mainstream school at the age of 13 years. A group of 7% stayed in primary and secondary level mainstream up to the age of 17. An equal group of 6% had never attended any school, they were in residential care or daycare.

**Figure 2:**
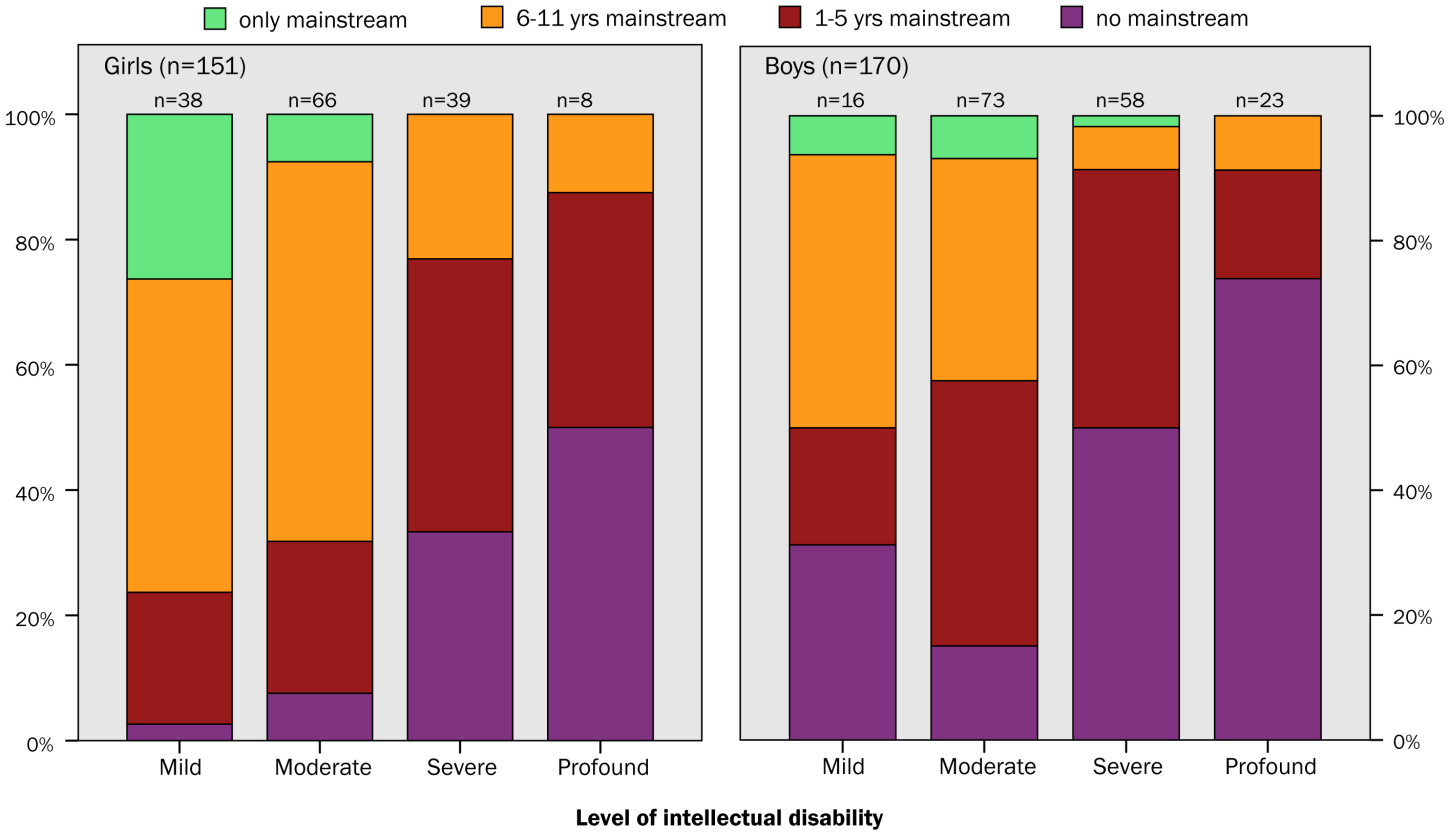
The proportion of resp. girls (n = 151) and boys (n = 170) in categories of years of mainstream school by level of intellectual disability.

Data on overall presence in mainstream primary school, enrolment at the age of 16 years and average enrollment from 4 to 16 years are presented in Table 2. A total of 74% of the children had entered mainstream. A majority (60%) was in mainstream for at least 3 years; girls more often and they stayed in longer. Boys were on average 5.5 years in mainstream, girls 7.0 years (*p* = 0.001). Boys were on average 8.9 years in special schools, girls 7.1 years (*p*<0.001). It is more relevant to state that 60% stayed in three years or longer, since the distribution of total years of mainstream showed these four peaks; no mainstream school at all, three years (range 1–5 years), 9 years (range 6–11 years) and a small peak at 13 years (12–13 years, i.e. only mainstream). Therefore we made these four subgroups: only mainstream which equals 12–15 years mainstream, 6–11 years, 1–5 years and no mainstream. Figure 2 shows the proportion of boys and girls in these four categories by level of intellectual disability. Since the group with only mainstream school was small, the group was combined with those who were 6–11 years in mainstream school (Table 3). Therefore ordinal regression analysis was conducted with duration of mainstream schooling in three groups as outcome. As few children with profound disability entered mainstream education, their duration in mainstream school was shorter, however not significant. Children with severe intellectual disability stayed shorter in mainstream school than those with a moderate disability (*p* = 0.021). Those with a mild intellectual disability did not significantly stay in longer than those with a moderate disability. Parental education and sex was not associated with duration in mainstream school.

**Table 2:** School enrolment* of Dutch children with Down syndrome (n = 319); arranged by sex.

	Total		Boys		Girls		
	n	%	n	%	n	%	*p*
*Overall enrolment in mainstream primary school*							
Ever in	237	73.6	108	63.5	129	84.9	<0.001
*Enrolled at 16 years of age*							0.002
Special School	276	86.5	142	84.5	134	88.7	
Mainstream Secondary School	23	7.2	8	4.8	15	10.0	
None	20	6.3	18	10.7	2	1.3	
*Average school enrollment in years* * *(mean ± SD)*							
Mainstream School (n = 237)	6.3±3.7		5.5±3.7		7.0±3.6		0.001
Special School (n = 285)	8.0±3.5		8.9±3.3		7.1±3.5		<0.001

*Data of school enrollment from 4 to 16 years were presented, since data on 17 and 18 years were not complete (some subjects are under 17 at the time of study).

**Table 3:** Univariate regression weights of the ordinal regression models in 16–19-year-olds with Down syndrome (n = 317]; positive weights indicate a higher probability of being longer in mainstream school (higher category of the outcome] compared to the reference.

		Unadjusted w° (95%CI]	Adjusted w† (95%CI]
Degree of Intellectual Disability∧	Mild	0.447 (−0.127,1.022]	0.182 (−0.328,0.693]
	Moderate (reference]	0	0
	Severe	−0.514 (−0.897,−0.132]**	−0.441 (−0.815, −0.068]*
	Profound	−0.289 (−1.073,0.495]	−0.229 (−0.981,0.522]
Parental Education	Low	−0.374 (−0.896,0.147]	−0.129 (−0.609,0.350]
	Medium	−0.098 (−0.475,0.280]	−0.018 (−0.347,0.312]
	High (reference]	0	0
Sex	Girls	0.330 (−0.013,0.674]	0.146 (−0.164, 0.456]
	Boys (reference]		0

∧ Degree of intellectual disability is based on all items of the SRZ.

° Unstandardized regression weights.

† Unstandardized regression weights adjusted for parental education and sex.

* *p*-value<0.05.

** *p*-value<0.001.

## Discussion

Most Dutch children with DS, 74%, participated socially and entered mainstream primary school. They entered at a slower rate, at the appropriate age of 4 years 53% had started mainstream primary school, another 10% a year later. At special school 10% started at the age of 4, another 6% at 5 years of age, another 13% at 6 years. Moreover, dropout rate from mainstream to special school was high at the age of 6–7 years. In the end of primary school only 17% was still in mainstream at the age of 13 years. Just a small group of 7% stayed in throughout secondary mainstream education. Overall most children stayed in mainstream school for 3 years or longer (60%). Girls were more often in mainstream school and stayed in longer. As expected, there is a significant association between the level of intellectual disability and years in mainstream school: those with a moderate disability stay in longer than those with severe intellectual disability. Parental education and sex did not contribute to this association.

Our study has some limitations. Selection bias may be present. Parents with a more positive attitude towards their child may be more inclined to join the parent organization or participate in this study. Conversely it is possible that those with relatively more problems may seek support by joining a parent organization or are inclined to participate in order to be able to express their problems. Institutionalized children could be underrepresented as parents might be unable to answer our questions. Also our data were obtained from questionnaires reported by parents retrospectively. However, the specific questions dealing with the type of school were directly answered or clarified in the comments (which made it easy to score missing data). We, therefore, think parents accurately answered such questions as Dolva also suggested [8]. We did not investigate the arguments of parents to choose for mainstream or special schooling, neither the actual possibility to be admitted to mainstream primary school, nor the reason to drop out. Some parents may not be able to find a mainstream school to admit their child with DS. Another limitation of our study is lack data on of academic performance. No information is available about repeating classes and degree of special support needed and whether this indeed had been offered. We studied the cohort up to 18 years, and report a 7% enrolment in mainstream secondary school, this is the likely lower estimate of children that has completed mainstream primary school with full academic levels. Up to the age of 13 years 17% attended mainstream primary school, the higher estimate of children with DS that completed mainstream primary. The results of our study gave a somewhat lower mainstream school participation compared to the estimates of De Graaf [6], who noticed a remarkable increase between 1984 and 2011. Because of differences in study design a close comparison cannot be made. Our cohort was born between 1992–1994, more recent year cohorts showed higher initial attendance rates of mainstream education.

The reason children with DS entered school after the age of 4 years may be due to their overall development. Dolva et al. [8] found that postponement of school entry was related to skills in self-care and social functioning. In Norway skills related to toileting, functional comprehension and expressive communication, problem solving and initiation of simple chores were lower at the age of 5 among children whose school entry was postponed compared to others. In the Netherlands mainstream schools refuse usually children not fully toilet trained. Other reasons for a late entry could be efforts to locate a school and willingness of the educational staff to accept a child with DS, time necessary to organize special facilities and parental hesitation to make the decision and formalize transition.

The majority of Dutch children started mainstream with a steady group opting out annually until the age of 13 years. At transition to secondary education more children transfer out into special education. Transition could be difficult as parents may reconsider the optimal place for their child at puberty. Secondary schools are clustered and some became large institutions with various levels and large amount of pupils. This requires social skills in order to travel to and from school independently. It seems only a fraction of adolescents with DS master such practical skills. The vast majority (90%) of adolescents with DS experience significant problems in social functioning [9]. Children with DS are socially vulnerable and may require a special setting especially before entering puberty [12].

More girls entered mainstream primary school than boys and they stayed in longer (on average 7.0 years, compared to 5.5 years for boys). This sex difference is significant, and has not been reported in other cohorts of children with DS. Only one study speaks of “a female advantage for verbal abilities in the DS population”, however this thesis has not been published [13]. It is not known what issues may attribute to the more favorable school career for girls with DS.

Also the high dropout rate needs to be confirmed. With all the efforts put into the enrolment of children with DS in mainstream school, some explanation of the high dropout needs to be studied. Is it merely an issue of cognitive and social development? The reported advantages of mainstream schooling for children with DS are more academic and language skills; especially some improvement in communication, expressive language or reading and literacy skills. To what extent are these effects of mainstream school enrolment on the overall development worthwhile? Adolescents with DS have limited abilities to perform relatively more complex tasks and experience serious difficulties in social functioning [14]. Serious problem behavior is also highly prevalent in them [9]. How could mainstream school improve effectively social functioning, behavior and the ability to perform more complex tasks? We hope future studies on mainstream school will also focus on overall social competence. We need to answer questions like; what determines a successful mainstream school career? what is the optimal education for individuals with DS? what is the most desirable curriculum? what long term goals do we need to set? Besides health, growth, development and cognition, pediatricians and other caretakers should discuss school choice in these perspectives. Their advice should aim to achieve optimal social competence and skills for independent living as well as the need for a focus on general behavior improvement and on the detection and treatment of specific psychopathology. Such an approach may improve the self-esteem of adolescents with DS. In this prospect school choice is relevant and should be discussed in wider perspective than just academic achievements.

### Conclusions

It is common for Dutch children with DS to enter mainstream school, 74% does. Most children stayed in 3 or more years, however, dropout rate was considerably. There is a sex difference in mainstream schooling in favor of girls. A significant association was found between level of intellectual disability and years in mainstream: those with a moderate disability stay in longer than those with severe intellectual disability.
